# An Observational Study of Clinical Bacteriological Profile and Antibiotic Susceptibility Pattern of Neonatal Sepsis at a Tertiary Care Hospital

**DOI:** 10.7759/cureus.111744

**Published:** 2026-06-29

**Authors:** Shreya Aggarwal, Samrudhi C Sonawane, Avinash L Sangle, Mohd Saeed Siddiqui, Madhuri B Engade, Sunil Gavhane

**Affiliations:** 1 Pediatrics, Mahatma Gandhi Mission's (MGM) Medical College and Hospital, a Constituent Unit of MGM Institute of Health Sciences, Aurangabad, IND

**Keywords:** antibiotic resistance (abr), culture-negative sepsis, culture-proven neonatal sepsis, klebsiella pneumoniae, neonatal intensive care unit (nicu)

## Abstract

Background: Neonatal sepsis is a significant cause of morbidity and mortality, especially in developing countries. Periodic assessment and documentation of clinical and bacteriological profiles and antibiotic susceptibility patterns of neonatal sepsis across geographic regions is essential to guide rational management.

Methodology: This observational, cross-sectional study was conducted at the neonatal intensive care unit (NICU) of Mahatma Gandhi Mission (MGM) Medical College and Hospital, Aurangabad, from September 2022 to June 2024. A total of 200 neonates between 28 and 42 weeks of gestation with either clinical or culture-proven sepsis were enrolled. Clinical and demographic data were collected using a structured proforma, and blood cultures were performed using standard microbiological methods. Antimicrobial susceptibility testing was conducted according to Clinical and Laboratory Standards Institute (CLSI) guidelines.

Results: Out of 200 neonates, 123 (61.5%) were males and 77 (38.5%) females, with the majority being preterm and low birth weight (LBW). Culture positivity was seen in 53 (26.5%) cases. *Klebsiella pneumoniae* ​​​​​was the predominant isolate found in 12 (22.6%) cases, followed by *Escherichia coli* in 6 (11.3%) cases. Gram-negative organisms exhibited high resistance to third-generation cephalosporins, while aminoglycosides and carbapenems showed the best sensitivity profile. Maternal risk factors such as anemia and fever were frequently associated with neonatal sepsis.

Conclusions: *Klebsiella pneumoniae* remains the leading cause of neonatal sepsis in this region, with worrying resistance trends to commonly used antibiotics. Continuous microbial surveillance and adherence to antibiotic stewardship protocols are vital to prevent treatment failures and improve neonatal outcomes.

## Introduction

Neonatal sepsis contributes significantly to mortality in the under-five-year age group. The clinical presentation, causative organisms, and susceptibility to antibiotics differ markedly across geographic regions [1-4]. The diagnosis of neonatal sepsis needs a high degree of suspicion, as the diagnosis is often delayed until there are advanced disease stages and ominous clinical findings. The diagnostic gold standard remains the blood culture, which has several limitations, including the long time required. The delay in confirmatory diagnosis underscores the importance of empirical antibiotic treatment. Empirical treatment requires knowledge of local patterns of organism involvement and antibiotic susceptibility [5-8]. Thus, there is a need for research studies describing the clinical profile, bacteriological data, and antibiotic susceptibility. The present study was undertaken to evaluate the following: (i) the clinical profile, especially birth weight, gestational age, mode of delivery, and blood culture reports; (ii) bacteriological profile, such as Gram-positive and Gram-negative bacteria isolated in culture-positive cases; and (iii) antibiotic susceptibility pattern of neonatal sepsis cases admitted to the neonatal intensive care unit (NICU) of a teaching hospital in Aurangabad, India.

## Materials and methods

This observational, cross-sectional study was conducted in the neonatal intensive care unit (NICU) at Mahatma Gandhi Mission (MGM) Medical College and Hospital, Aurangabad, Maharashtra, India, from September 2022 to June 2024. Ethical committee approval for the study was obtained from the Institutional Ethics Committee of MGM Medical College and Hospital (approval number: MGM/PHARMAC/ECRHS/2022/134). Informed written consent was obtained from the parents or guardians of all participating neonates. All patient data were anonymized, and confidentiality was strictly maintained throughout the study.

All neonates aged 0-28 days who were admitted to the NICU with either clinical suspicion or culture-proven sepsis were included after obtaining informed parental consent. Data were collected over 18 months, from January 2023 to June 2024, following ethics approval. The study duration restricted the sample size. Neonates presenting with clinical features suggestive of sepsis, such as fever, lethargy, respiratory distress, poor feeding, jaundice, convulsions, or hypothermia, and those with positive blood culture results confirming sepsis were eligible for inclusion [2]. Exclusion criteria comprised neonates with congenital malformations, chromosomal abnormalities, or dysmorphic features; those born to human immunodeficiency virus (HIV), hepatitis B surface antigen (HBsAg), or Venereal Disease Research Laboratory (VDRL)-positive mothers; and neonates who had received antibiotic treatment for more than 48 hours before NICU admission. A total of 200 neonates satisfied the inclusion criteria and were analyzed. Data were collected prospectively using a predesigned proforma from NICU records and patient case files. In an aseptic environment, 1 milliliter of venous blood was collected in two blood culture bottles and processed using the BACT/ALERT system. Two datasets were maintained for analysis. The first dataset contained demographic, perinatal, and clinical details, including age at presentation, sex, birth weight, gestational age, and mode of delivery. Maternal factors such as fever and anemia were recorded. Outcomes such as hospital stay duration and discharge or mortality status were also noted.

The second dataset included only those neonates with positive blood culture reports. It detailed the type of bacterial isolates and the corresponding antibiotic susceptibility patterns. Antibiotic susceptibility testing was performed using the Kirby-Bauer disk diffusion method on Mueller-Hinton agar, in accordance with the Clinical and Laboratory Standards Institute (CLSI) 2023 guidelines. The antibiotic panel tested comprised aminoglycosides (amikacin and gentamicin), β-lactams (cefotaxime, ceftazidime, and ceftriaxone), carbapenems (meropenem and imipenem), penicillin (piperacillin-tazobactam and ampicillin), and other agents, such as vancomycin, linezolid, ciprofloxacin, and colistin.

For this study, clinical sepsis was defined as the presence of two or more clinical signs of infection, with or without a positive blood culture. Culture-positive sepsis was defined as laboratory-confirmed bacteremia, as evidenced by a positive blood culture report. Early-onset sepsis (EOS) refers to infection occurring within 72 hours of life. It was presumed to be maternally acquired, whereas late-onset sepsis (LOS) was defined as infection occurring after 72 hours of life, typically due to nosocomial or community-acquired sources.

Data from both master charts were entered into Microsoft Excel 2021 (Microsoft Corp., Redmond, WA). Continuous variables were expressed as mean ± standard deviation (SD), while categorical variables were summarized as frequencies and percentages.

## Results

A total of 200 neonates were included in the study over an 18-month period. Male neonates predominated with 123 (61.5%) cases, and most deliveries were via lower segment cesarean section, noted in 129 (64.5%) cases. Culture positivity was observed in 26.5% (n=53) of neonatal sepsis cases. The most common maternal risk factors were maternal anemia observed in 52 (26%) cases and maternal fever in 51 (25.5%) cases. Regarding neonatal characteristics, 99 (49.5%) neonates had normal birth weight, while 76 (38%) neonates were low birth weight (LBW). Early-term gestation (37-38 weeks) accounted for 149 (74.5%) cases (Table 1).

**Table 1:** Demographic and Clinical Profile of Neonates LSCS: lower segment cesarean section

Variable	Category	Number (%)
Gender	Male	123 (61.5)
	Female	77 (38.5)
Mode of delivery	LSCS	129 (64.5)
	Vaginal delivery	71 (35.5)
Sepsis onset	Early-onset sepsis (≤72 hours)	98 (49)
	Late-onset sepsis (>72 hours)	102 (51)
Sepsis classification	Clinical sepsis	147 (73.5)
	Culture-positive sepsis	53 (26.5)
Birth weight (g)	Normal (2,500-3,500)	99 (49.5)
	Low birth weight (1,500-2,499)	76 (38)
	Very low birth weight (1,000-1,499)	21 (10.5)
	Extremely low birth weight (<1,000)	4 (2)
Gestational age (weeks)	Early term (37-38)	149 (74.5)
	Full term (39-40)	31 (15.5)
	Preterm (28-36)	20 (10)
	Extreme preterm (<28)	1 (0.5)
Maternal risk factors	Maternal anemia	52 (26)
	Maternal fever	51 (25.5)
	Chorioamnionitis	33 (16.5)
	Surgical intervention during pregnancy	45 (22.5)
	History of COVID-19	20 (10)

Gastrointestinal manifestations were the most common clinical presentation noted in 182 (91%) cases, followed by respiratory manifestations in 143 (71.5%) cases and central nervous system involvement in 116 (58%) cases. Respiratory support was required in 146 (73%) cases via O₂ hood or prongs. Invasive interventions included feeding tubes, umbilical lines, and peripherally inserted central catheter (PICC) lines. Kangaroo Mother Care was utilized in 168 (84%) neonates. Cerebrospinal fluid (CSF) analysis in suspected meningitis cases showed mean protein levels of 123.6±90.5 mg/dL. The overall mortality rate was 4.5% (n=9), with 77.5% (n=155) of neonates discharged successfully (Table 2).

**Table 2:** Clinical Manifestations and Laboratory Profile SD: standard deviation, CSF: cerebrospinal fluid, PICC: peripherally inserted central catheter

Parameter	Finding	Value/number (%)
Clinical systems involved	Gastrointestinal	182 (91)
	Respiratory	143 (71.5)
	Central nervous system	116 (58)
	Cardiovascular	99 (49.5)
Respiratory support	O₂ hood/nasal prongs	146 (73)
	Invasive/non-invasive ventilation	66 (33)
Interventions	Feeding tube required	170 (85)
	Umbilical line	67 (33.5)
	PICC line	52 (26)
	Kangaroo Mother Care	168 (84)
Laboratory markers (mean±SD) (day 1)	Hemoglobin (g/dL)	17.5±2.6
	Total leukocyte count (/cmm)	12,248±6,714
	Platelet count (×10³/μL)	211,010±122,277
	C-reactive protein (mg/L)	11.7±16.8
CSF analysis (mean±SD)	Glucose (mg/dL)	49.9±14.7
	Proteins (mg/dL)	123.6±90.5
	Cells (cells/mm³)	8.1±16.3
Outcomes	Discharged	155 (77.5)
	Discharge against medical advice	36 (18)
	Mortality	9 (4.5)

Among the 53 culture-positive isolates, Gram-negative bacteria were predominant, noted in 34 (64.2%) isolates, compared to Gram-positive organisms in 15 (28.3%) isolates and fungi in 4 (7.5%) cases. *Klebsiella pneumoniae* was the most frequently isolated pathogen, followed by *Acinetobacter baumannii* and *Escherichia coli*. Among Gram-positive isolates, *S. haemolyticus* was the leading cause. Fungal sepsis was caused by *Candida* species in four (7.5%) cases (Table 3).

**Table 3:** Bacteriological Profile MRSA: methicillin-resistant *S. aureus*

Organism type	Pathogen	Number	% of isolates
Gram-negative bacilli	Klebsiella pneumoniae	12	22.6
	Acinetobacter baumannii	7	13.2
	Escherichia coli	6	11.3
	Pseudomonas aeruginosa	3	5.7
	Serratia marcescens	2	3.8
	Enterobacter cloacae	2	3.8
	Acinetobacter haemolyticus	1	1.9
	Enterobacter aerogenes	1	1.9
	Total Gram-negative	34	64.2
Gram-positive cocci	Staphylococcus haemolyticus	5	9.4
	Staphylococcus epidermidis	2	3.8
	Staphylococcus hominis	2	3.8
	Enterococcus faecalis	1	1.9
	MRSA	1	1.9
	*Streptococci* species	4	7.5
	Total Gram-positive	15	28.3
Fungi	*Candida* species	4	7.5
Total isolates		53	100

Gram-negative isolates demonstrated high susceptibility to carbapenems, with meropenem showing the highest sensitivity. Colistin, ciprofloxacin, and cefepime also showed high efficacy. However, sensitivity to first-line agents such as ceftriaxone and amoxicillin-clavulanic acid was lower. Among Gram-positive isolates, levofloxacin and tigecycline showed higher sensitivity compared to vancomycin and linezolid. Notably, specific multidrug-resistant (MDR) pathogens such as *K. pneumoniae* and *A. baumannii* exhibited variable resistance patterns to colistin and piperacillin-tazobactam (Table 4).

**Table 4:** Antibiotic Susceptibility Pattern

Antibiotic class	Agent	Gram-positive sensitivity (%) (n=15)	Gram-negative sensitivity (%) (n=34)
Aminoglycosides	Gentamicin	9 (60%)	32 (94.1%)
	Amikacin	1 (6.7%)	32 (94.1%)
β-lactams	Ampicillin	3 (20%)	1 (6.7%)
	Amoxicillin-clavulanic acid	-	21 (61.8%)
	Ceftriaxone	3 (20%)	21 (61.8%)
	Cefepime	1 (6.7%)	32 (94.1%)
	Piperacillin-tazobactam	1 (6.7%)	30 (88.2%)
	Cefoperazone-sulbactam	1 (6.7%)	32 (94.1%)
Carbapenems	Meropenem	1 (6.7%)	33 (97.1%)
	Imipenem	1 (6.7%)	32 (94.1%)
Fluoroquinolones	Ciprofloxacin	12 (80%)	32 (94.1%)
	Levofloxacin	12 (80%)	14 (41.2%)
Glycopeptides/others	Vancomycin	6 (40%)	1 (6.7%)
	Linezolid	6 (40%)	1 (6.7%)
	Tigecycline	12 (80%)	24 (70.6%)
	Colistin	1 (6.7%)	32 (94.1%)
	Cotrimoxazole	9 (60%)	32 (94.1%)

## Discussion

Among the 200 neonates, male predominance was noted. Culture positivity was observed in 26.5% (n=53) of neonatal sepsis cases. Among the 53 culture-positive isolates, Gram-negative bacteria were predominant, compared to Gram-positive organisms and fungi. *Klebsiella pneumoniae* was the most frequently isolated pathogen, followed by *Acinetobacter baumannii* and *Escherichia coli*. Among Gram-positive isolates, *S. haemolyticus* was the leading cause. Gram-negative isolates demonstrated high susceptibility to carbapenems. Colistin, ciprofloxacin, and cefepime also showed high efficacy. However, sensitivity to first-line agents such as ceftriaxone and amoxicillin-clavulanic acid was lower. Among Gram-positive isolates, levofloxacin and tigecycline showed high sensitivity.

Fleischmann et al. reviewed the global incidence and clinical aspects of neonatal sepsis and reported that one-third of cases show culture positivity. *Staphylococcus aureus* and *Klebsiella* species were noted as the most common causative organisms. However, like our study results, the low number of isolates limits the validity of reported aetiology, and the study authors also highlighted the lack of data regarding antibiotic susceptibility patterns in research studies and the need for more epidemiologic data [4]. De Rose et al. stressed the extreme susceptibility of newborns to life-threatening sepsis and the need for data from the local geographic area regarding causative organisms, clinical features, and antibiotic susceptibility. The study authors suggested the use of ampicillin and gentamicin as the first-choice antibiotic therapy for empirical treatment of early-onset sepsis [9]. Wen et al., in line with our study observations, found that Gram-negative bacteria were responsible for significant cases of neonatal sepsis in lower-middle-income countries [10]. Jatsho et al. from Bhutan reported that Gram-negative organisms were more frequently isolated, and *Klebsiella pneumoniae* was the most common organism. Like our study findings, there was a high prevalence of antibiotic resistance to first-line antibiotics like ceftriaxone. The need for antibiotic stewardship was mentioned [11]. Yadav et al. reported that there were 16.9% cases of neonatal sepsis that showed positive blood culture. *Staphylococcus aureus* (35.6%) and *Klebsiella pneumoniae* (15.3%) were the commonly isolated organisms, and meropenem and imipenem showed the highest 100% efficacy, while cefotaxime was least (28%) effective among Gram-negative isolates [12]. Islam et al. observed that *Acinetobacter* was the most common isolated organism, and a 10-day regimen of antibiotics was comparable to a two-week antibiotic regimen [13]. Pataskar et al. from Maharashtra in India found that respiratory distress was a common presentation observed in 52.2% of neonatal sepsis cases. *Klebsiella* spp. (36.7%) and *Acinetobacter* spp. (31.1%) were the common organisms isolated, and the mortality rate was 30%. It was suggested that empiric antibiotic therapy should provide coverage against *Klebsiella* and *Acinetobacter* microbes [14]. Sturrock et al. reported the lacunae in neonatal sepsis management in resource-limited settings and gave recommendations to overcome them. It was highlighted that despite frequent over-the-counter treatment in South Asia, there was low antibiotic resistance in the community setting as compared to in-hospital treatment. It was recommended that clinicians should use first-line antibiotics in the community setting as far as possible and avoid exposure of neonates to multidrug-resistant organisms in the hospital setting [15].

The study's limitations are its descriptive study design and a restricted sample from a single tertiary care center. The study had fewer culture-positive cases, and the hospital setting introduced selection bias. The limitations affect the external validity of the study's data. More studies on the epidemiological aspects of neonatal sepsis, especially causative organisms and antibiotic susceptibility patterns, are needed.

## Conclusions

The neonatal sepsis cases in our study had a male predominance, and gastrointestinal and respiratory complaints were more commonly observed in them. About one in four neonatal sepsis cases had a positive blood culture, and Gram-negative bacteria were predominant. *Klebsiella* was the most frequently isolated pathogen, followed by *Acinetobacter* and *Escherichia coli*. Carbapenems, fluoroquinolones, colistin, and cefepime were highly effective against isolated organisms. Although our study had limited single-center data, empiric treatment should take into account the bacteriological profile and antibiotic susceptibility patterns reported in the regional literature.
